# New insights into GPCR coupling and dimerisation from cryo-EM structures

**DOI:** 10.1016/j.sbi.2023.102574

**Published:** 2023-06

**Authors:** Anastasiia Gusach, Javier García-Nafría, Christopher G. Tate

**Affiliations:** 1 MRC Laboratory of Molecular Biology, Francis Crick Avenue, Cambridge CB2 2QH, UK; 2 Institute for Biocomputation and Physics of Complex Systems (BIFI) and Laboratorio de Microscopías Avanzadas (LMA), University of Zaragoza, 50018, Zaragoza, Spain

## Abstract

Over the past three years (2020–2022) more structures of GPCRs have been determined than in the previous twenty years (2000–2019), primarily of GPCR complexes that are large enough for structure determination by single-particle cryo-EM. This review will present some structural highlights that have advanced our molecular understanding of promiscuous G protein coupling, how a G protein receptor kinase and β-arrestins couple to GPCRs, and GPCR dimerisation. We will also discuss advances in the use of gene fusions, nanobodies, and F_ab_ fragments to facilitate the structure determination of GPCRs in the inactive state that, on their own, are too small for structure determination by single-particle cryo-EM.

## Highlights

• Promiscuous GPCR coupling shows mechanism of secondary coupling.
• Cryo-EM structures of GPCR dimers have been determined for Class A, C and D receptors.
• The first GPCR-GRK structure has been determined.
• Arrestin-coupled GPCR structures provide new insights for the development of biased agonists.
• Methodologies have been developed for determining cryo-EM structures of inactive state GPCRs.

## Graphical abstract

**Figure:**
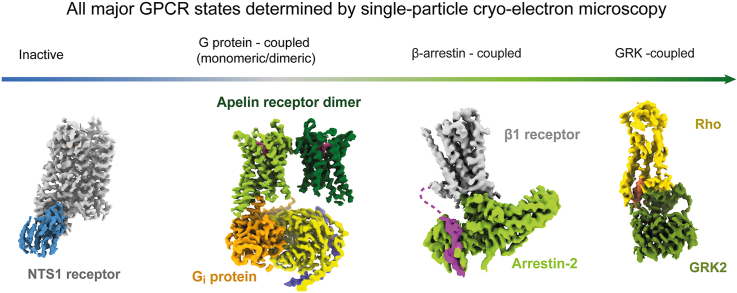

## Introduction

GPCRs play a pivotal role in intercellular signalling throughout the human body and are the targets of 34% of FDA approved drugs [1]. Only a proportion of all GPCRs have been drugged and there is intense scrutiny of other GPCRs to develop novel therapeutics for the treatment of diseases such as diabetes, cancer and neurodegeneration [2]. Structural biology plays a key role in drug development through either providing a structure suitable for screening *in silico* ultra-large drug libraries [3••] or through providing a mechanistic understanding of fundamental molecular processes such as receptor and G protein activation [4,5]. Here we highlight a few of the fundamental molecular insights that underpin complexities in GPCR pharmacology that have been uncovered by the wealth of structures determined by cryo-EM over the past few years.

## Structural mechanisms in promiscuous GPCR-G protein coupling

GPCRs signal through heterotrimeric G proteins and the type of α-subunit determines the downstream signalling cascade affected. There are four major families of G proteins in humans, G_s_, G_i/o_, G_q/11_ and G_12/13_ that signal through different pathways. Although some GPCRs are specific and activate a single type of G protein, at least 50% of GPCRs activate two or more G proteins [6, 7, 8]. Promiscuous coupling activates different G proteins with varying efficacies and kinetics, generating a fingerprint-like signalling profile within the cell [9], thus enhancing the complexity of GPCR signalling and providing new therapeutic opportunities.

Cryo-EM structures of eleven GPCRs have been determined with each GPCR coupled to two or more distinct G proteins: GCGR, β_1_AR, ADGRF1 and 5HT_4_R coupled to G_s_ and G_i/o_ [10, 11, 12, 13], NK_1_R coupled to G_s_ and G_q/11_ [14], CCK_A_R coupled to G_q_, G_i1_ and G_s_ [15,16], ADGRL3 coupled to G_s_, G_i_, G_q_ and G_12_ [17••] and four receptors coupled to G_i/o_ and G_q/11_ (GSHR [18,19], CCK_B_R [20], GPR139 [21] and MRGPRX2 [22]). Several trends arise from analysing this set of structures [23].

**Figure 1:**
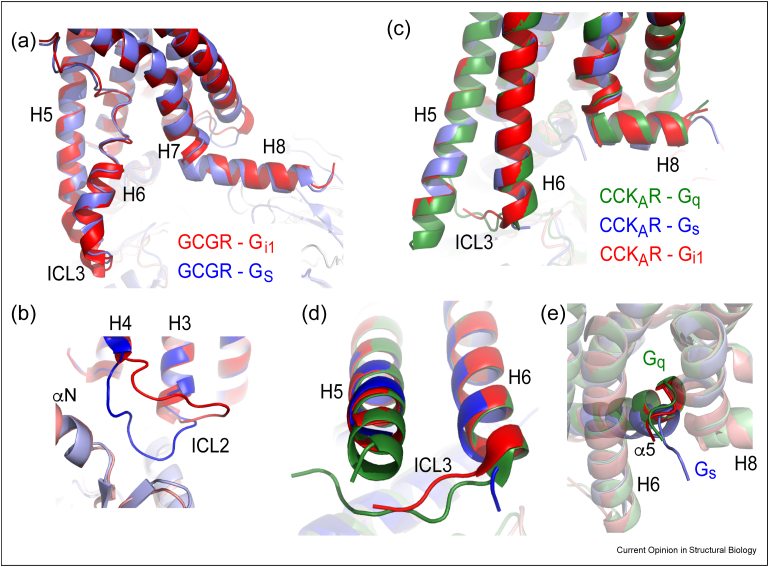
Structural snapshots of promiscuous GPCR-G protein coupling. Structural superposition of the GCGR coupled to G_s_ (blue) and G_i1_ (red) showing similarities in TM6 **(a)** and differences in ICL2 **(b)** [10]. Structural superposition of the CCK_A_R coupled to G_q_ (green), G_i1_ (red) and G_s_ (blue) showing similarities in TM6 position **(c),** differences in the ordering of ICL3 depending on the coupled G protein **(d)** and differences in the engagement mode of the α-subunit C-terminal ‘wavy hook’ for G_s_ vs G_q_**(e)** [15,16].

The outward movement of the cytoplasmic end of transmembrane helix TM6 is a hallmark of GPCR activation and is thought to determine the size and shape of the intracellular cleft where the cytoplasmic end of helix α5 of the G protein α-subunit couples [24]. Structures of many different GPCRs coupled to G proteins suggested initially that the magnitude of TM6 displacement correlated with the type of G protein. A large outward movement of TM6 forms a wide intracellular cleft that is required typically for G_s_ coupling, whilst smaller movements of TM6 form a narrower cleft characteristic of G_i/o_-G_q/11_ coupling [25,26]. However, recent new structures show that this is not always the case when they are the secondary couplers, with G_s_ sometimes coupling to a narrow cleft and G_i_ or G_q_ coupling to a wide cleft. Structures of the same GPCR coupled to either G_s_ or another G protein suggest that the movement of TM6 is usually the same regardless of the secondary G protein coupled *i.e.* the secondary G protein has to use a similar intracellular cleft for coupling as the primary G protein (Figure 1a–e). For example, the primary coupler to GCGR is G_s_ and the GCGR-G_s_ cryo-EM structure shows a wide intracellular cleft; the receptor structure coupled to its secondary coupler G_i/o_ shows an equally wide cleft to when G_s_ is coupled, and not a narrow cleft as might be expected [10]. Conversely, CCK_A_R and NK_1_R couple primarily to G_q_ and adopt a narrow intracellular cleft upon activation, and the secondary G protein G_s_ also couples to this narrow cleft. In some instances, such as for CCK_A_R, this forces the G protein to adopt ‘non-standard’ conformations where the α-subunit shows an unwinding of the ‘wavy hook’ in the α5 helix C-terminus, which protrudes outwards from the receptor intracellular cavity (Figure 1e). Primary coupling of G_i/o_ and G_q/11_ results in a similar narrow intracellular cleft, which may explain the high abundance of G_i/o_-G_q/11_ promiscuous couplings [7].

The intracellular loops (ICLs) of GPCRs are the elements that differ most when coupling to different G proteins. However, there appears to be no correlation with the type of ICL rearrangement and the type of G protein or primary/secondary couplings. ICL3 takes a prominent role in promiscuous G protein coupling in MRGPRX2, 5-HT_4_R, ADGRF1, GSHR, GPR139, and CCK_A_R where it makes different interactions to different G proteins (Figure 1d.) ICL2 also changes conformation or interactions in most GPCR-G protein complexes (e.g. GCGR and GSHR, Figure 1b), whereas ICL1 differential interactions have only been observed in GCGR. The loop between TM7 and H8 also varies in β_1_AR coupled to either G_s_ or G_i_. Such differences in ICLs contribution to promiscuous G protein coupling were supported by mutagenesis and functional assays, where alterations in the CCK_A_R ICL3 had a major impact on G_q_ but not G_S_ or G_i_ signalling [15]. Similarly, alterations in the GCGR ICL3 and ICL1 showed a greater impact on G_i_ compared to G_S_ signalling [10].

## GPCR structures coupled to GRK or arrestin

One mechanism in the cell to terminate GPCR-G protein signalling at the plasma membrane is through receptor phosphorylation by GRKs, recruitment of arrestin via the phosphorylated C-terminus/ICL3 and then clathrin-mediated endocytosis mediated by arrestin-clathrin/AP2 interactions [27]. Arrestin interacts with GPCRs in two distinct ways. Arrestin binds first to the phosphorylated C-terminus/ICL3 of the receptor, causing a conformation change in arrestin that subsequently facilitates coupling of arrestin to the receptor [28, 29, 30]. Arrestin couples to GPCRs using the same intracellular cleft that binds the C-terminal α5 helix of the G protein [31] and results in activation of the intracellular ERK1/2 signalling cascade. It is crucial to understand the molecular differences between coupling of G proteins, GRKs and arrestins, because the therapeutic effect and side effects of drugs may arise through different signalling pathways [32]. There is thus intense interest in developing biased ligands that specifically activate/inhibit only one specific pathway.

**Figure 2:**
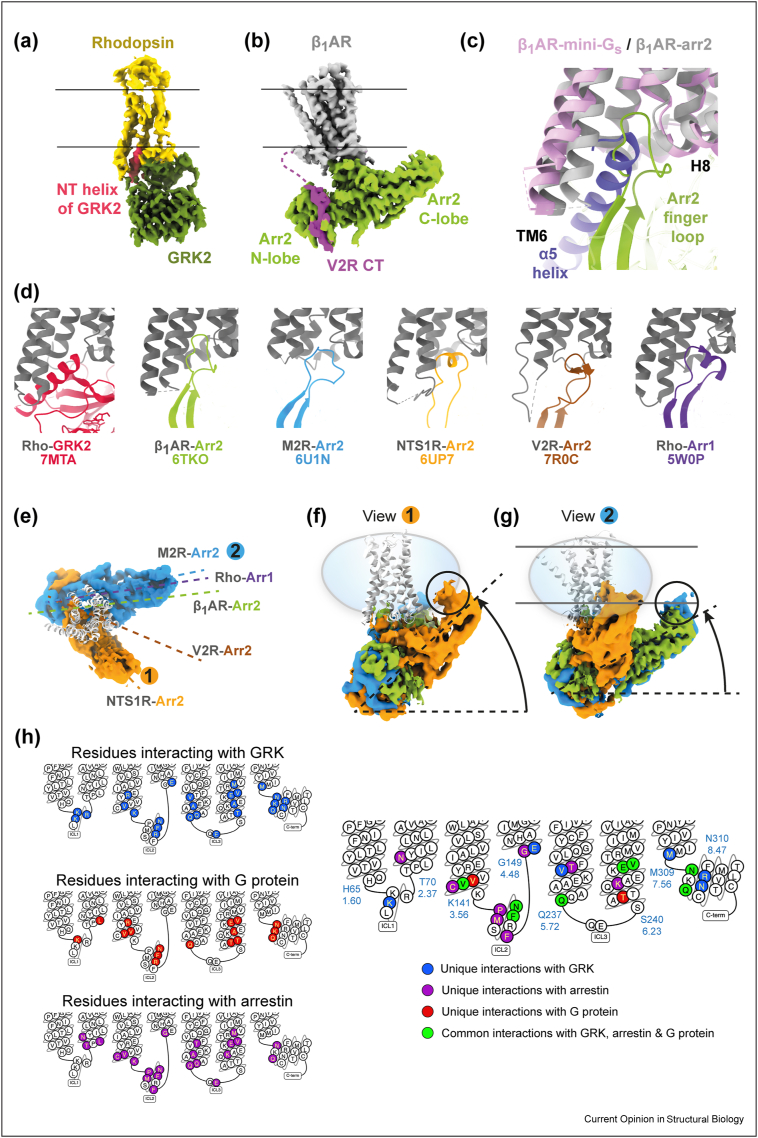
Variations in coupling of arrestins and GRK2 to GPCRs. **(a)** Cryo-EM density (EMDB-23979) of rhodopsin coupled to GRK1 [33]. Density for the F_ab_ required for structure determination has been removed for clarity. **(b)** Cryo-EM density of β_1_AR in a lipid nanodisc coupled to β-arrestin1 (EMDB-10515) [37]. Density for F_ab_30 required for structure determination has been removed for clarity. **(c)** Superposition of β_1_AR coupled to mini-G_s_ (purple; PDB code 7JJO [72]) and β_1_AR (grey) coupled to β-arrestin1 (green; PDB code 6TKO [37]). **(d)** Different conformations of the GRK coupling helix and arrestin finger loop when coupled to different receptors. **(e**–**g)** Variation in the angle of arrestin coupled to different receptors (see main text for references): **(e)** a view perpendicular to the membrane plane; panels **(f**–**g)** are views parallel to the membrane plane in positions 1 and 2, respectively, as defined in panel **(e)**. **(h)** Snake plots of bovine rhodopsin with amino acid residues within 3.9 Å (inclusive) of either GRK, G protein or arrestin coloured appropriately. PDB codes for the complexes are as follows: rhodopsin-GRK, 7MTB [33••]; rhodopsin-G protein, 6OYA [73]; rhodopsin-arrestin, 5W0P [74]. The panels were made using GPCRdb [75].

Structure determination of a GPCR-GRK complex has been difficult, however, stabilisation of the rhodopsin-GRK1 complex by a combination of crosslinking, binding of two F_ab_s and lipids resulted in the first low resolution structures [33••]. The receptor was in its active state, with the N-terminus of GRK1 forming an α-helix that binds to the intracellular cleft like G proteins and arrestin (Figure 2a,d). Comparison between the conformation of rhodopsin when coupled to either GRK, arrestin or the G protein transducin shows that they are virtually identical (RMSDs of 0.9–1.0 Å) and that the binding sites on rhodopsin overlap significantly (Figure 2h). There are eight residues that interact with all three coupled proteins (Val139^3.54^, Asn145^34.53^, Phe146^34.54^, Gln237^5.72^, Glu249^6.32^, Val250^6.33^, Asn310^8.47^, Gln312^8.49^) and a further subset of residues (Figure 2h) that interact only with GRK1 (6 residues), visual arrestin (8 residues) or transducin (2 residues).

Seven structures of GPCRs coupled to arrestins have now been determined. The first high-resolution structure of a GPCR-arrestin complex was a crystal structure of constitutively active mutant of human rhodopsin fused to a preactivated form of mouse arrestin 1 (visual arrestin) [34]. A variety of different strategies were required for cryo-EM structure determination of non-visual arrestins coupled to activated receptors, including combinations of the following: fusion with the C-terminus of phosphorylated V_2_ receptor, arrestin mutants, cross-linking, binding of F_ab_30 to stabilise the active state of arrestin and the use of lipid-mimicking environments. Structures of complexes with arrestin 2 (Arr2; also called β-arrestin1; Figure 2b,c) were determined coupled to NTS_1_R [35•,^36^], β_1_AR [37••], M_2_R [38•], V_2_R [39•] and 5HT_2B_R [40••].

G proteins couple to different receptors in a relatively conserved way [25], but in contrast arrestins have shown a wide variation in their binding poses. Significant variations occur in the structure of the finger loop of arrestin inserted into the intracellular cleft of the receptor (Figure 2d) and the angle of interaction between arrestin and the GPCR when viewed both perpendicular to the membrane plane and parallel to the membrane plane (Figure 2e–g).

Two GPCR-arrestin structures (β_1_AR [37] and 5HT_2B_R [40]) have been determined at 3.3 Å resolution where there is good density for the ligand in the orthosteric binding pocket. Comparison with the receptor bound to the same ligand but coupled either to a G protein (5HT_2B_R) or a G protein-mimetic nanobody (β_1_AR) showed similar weakening of interactions between the ligand and H5, explaining the weaker ligand affinity in the arrestin-coupled state compared to the G protein coupled state. There are also other differences between a G protein-coupled receptor and arrestin-coupled receptor, the most obvious one being the difference in outward movement of H6, although in β_1_AR this is less than in the G protein coupled state whereas for 5HT_2B_R it is greater than in the G protein coupled state. The differences observed between structures could be used in the development of biased agonists.

## GPCR dimers

The existence and functional role of obligate class C and class D GPCR dimers are well-established, both structurally and functionally [41,42]. However, for Class A receptors there is no consensus on whether dimerisation is a ubiquitous mechanism in regulating Class A GPCR function. Some class A GPCRs are accepted to form transient dimers and higher order oligomers, although their physiological role is often uncertain [43,44]. Any structural dimer composed of parallel protomers observed in either X-ray crystal structures [45] or cryo-EM has the potential to be physiologically relevant, but careful validation is required by biochemistry and pharmacology to support this.

**Figure 3:**
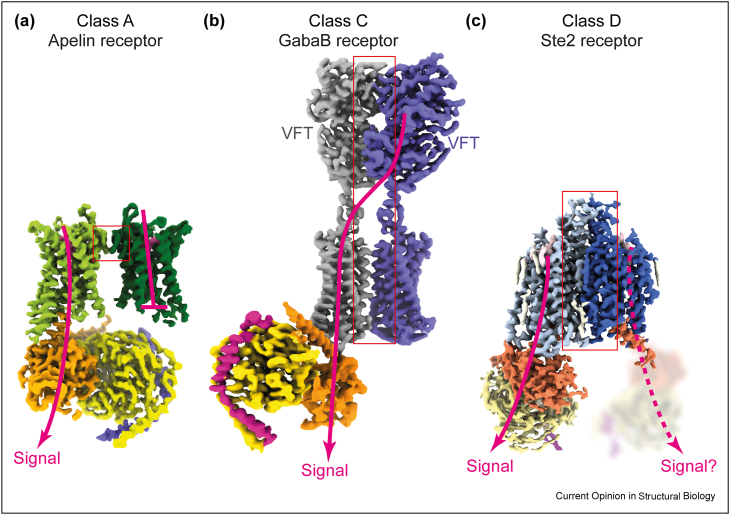
Signalling routes in GPCR dimers. **(a)** Cryo-EM density of the apelin receptor (EMDB-32243) shows that there is sufficient room for only one G protein to couple per dimer, and the C-terminus of the adjacent protomer binds in the G protein-coupling cleft in an auto-inhibitory mechanism [52]. The dimer interface is shown by in the GABAB receptor dimer (EMDB-21534) is from the VFT domain of one protomer through the transmembrane helices of the adjacent protomer that can couple to G protein. The structures of two transmembrane helical bundles are not identical and the G protein coupling site forms only in one protomer [46,47]. The dimer interface is shown by the red box. **(c)** The Ste2 dimer (EMDB-11720) contains two protomers of identical conformation that are both capable of coupling to G proteins simultaneously, although one G protein is highly mobile, with the exception of the α5 helix that is ordered where it contacts the receptor [53]. The tilt of the G protein with respect to the receptor is over 50° different from that observed in G protein-coupling to Class A receptors, thus allowing two G proteins to couple simultaneously. The signalling pathways through the receptor are assumed to follow the paradigm of a monomeric receptor, however it is unclear whether both G proteins can signal to the same extent and there could be crosstalk between protomers across the dimer interface [54]. The dimer interface is shown by the red box.

Humans possess 22 Class C GPCRs and there are now 76 cryo-EM structures, determined between 2019 and 2022, bound to either antagonist, agonist, positive allosteric modulator (PAM), negative allosteric modulator (NAM), regulator of G protein signalling (RGS) protein and/or G protein. Due to space constraints, we will discuss only those receptors where a fully active G protein-coupled state has been determined (Table 1), namely the GABA_B_ receptor [46••,^47^•] and metabotropic glutamate receptors (mGluRs) [48•]. The common feature of Class C dimers is that they are maintained dimeric predominantly through interactions in the extracellular Venus fly trap domain (VFT; Figure 3b) that binds agonists. The agonist-induced conformational change in the VFT is transmitted via a linker region to the transmembrane regions, ultimately resulting in a rotation of one helical bundle with respect to the other. In the GABA_B_ receptor, this changes the dimer interface from being formed by predominantly H5-H5 to H6-H6 [46••,^47^•] and in the mGluRs from mainly H4-H4 to H6-H6 [48•]. A number of variations between these states have also been described, highlighting the plasticity of these receptors and a number of different solutions for how PAMs can promote the formation of active-like states [46••,^48^•,^49^,^50^•]. Extensive pharmacological and biochemical studies have determined that only one protomer in the dimer couples to a G protein and that signalling is transmitted from the VFT of one receptor in the dimer to the G protein coupling site on the adjacent dimer [41]. This is recapitulated in the asymmetric active-state dimer structures where only a single G protein is coupled per dimer, via a coupling site formed through interactions primarily to ICL2, which is distinct to that found in other GPCR families [47•,^48^•,^51^•].

**Table 1:** Details of GPCR structures discussed in the main text.

Receptor		Dimer type	Class	PDB	Agonist (Ag), antagonist (Ant), PAM, NAM	G protein family	Stabilising antibodies and fusions	Reference
Apelin		Homo	A	7W0N	Ag	G_i_	scFv16 + BRIL	[52••]
				7W0L	Ag	G_s_	scFv16 +BRIL	
Ste2		Homo	D	7AD3	Ag	Gpa1	–	[53••]
				7QB9	–	–	–	[54••]
				7QA8	Ant	–	–	
				7QBC	Ag	–	–	
				7QBI	Ag	–	–	
GABA_B_		Hetero	C	7EB2	Ag	G_i_	scFv16	[47•]
			C	7CA3	PAM	–	–	[49]
			C	7CA5	–	–	–	
			C	7CUM	Ant + NAM	–	–	
			C	6UO8	Ag + PAM	–	–	[50•]
			C	6UO9	Ag	–	–	
			C	6UOA	Ag	–	–	
			C	6VJM	APO	–	–	
			C	7C7S	Ant	–	–	[46••]
			C	7C7Q	Ag + PAM	G_i1_	–	
			C	6WIV	–	–	–	[55]
			C	6W2X	Ant + NAM	–	–	[56]
		Homo	C	6W2Y	Ant + NAM	–	–	
Metabotropic glutamate receptors	mGlu1	Homo	C	7DGD	–	–	–	[57]
				7DGE	Ag		Nb43	
	mGlu2		C	7E9G	Ag + PAM	G_i_	scFv16 + Nb	[48•]
	mGlu2	Homo	C	7MTQ	Ant	–	–	[51•]
			C	7MTR	Ago-PAM + Ag	–	–	
			C	7MTS	Ago-PAM	G_i_		
	mGlu2	Homo	C	7EPA	–	–	–	[58•]
				7EPB	Ag		Nb-RON	
	mGlu7	Homo	C	7EPC	–	–	–	
	mGlu2mGlu7	Hetero	C	7EPD	–	–	–	
	mGlu5	Homo	C	6N52	–			[59]
		Homo		6N51	Ag		Nb43	
	mGlu5-5M	Homo	C	7FD8	Ag	–	–	[60]
		Homo	C	7FD9	Ant			
	mGlu3	Homo	C	7WI8	Ant	–	–	[61]
				7WI6	Ag + NAM	–	–	
				7WIH	Ag	–	–	
	mGlu4	Homo	C	7E9H	Ag	G_i3_	scFv16	[48•]

In contrast to Class C receptors, the cryo-EM structure of the class D receptor homodimeric GPCR Ste2 (Figure 3c) showed that it couples to two G proteins simultaneously [53••]. The density for one G protein was well-resolved, but the density for the adjacent G protein was diffuse and molecular dynamics simulations showed that each G protein underwent phases of mobility, with only one G protein being ordered at any one time. The interface between the two protomers is also dynamic [54••], even though it has a very large surface area in the active state (2500 Å^2^) and is composed of interactions between the N-terminus, ECL1 and H1. Cryo-EM structures of five different receptor conformations showed that Ste2 activation upon binding the native agonist α-factor involved an increase in the strength of the interface and a 20 Å movement of the cytoplasmic end of H7 [54••]. The movement of H7 unblocked the G protein coupling site and then formed additional contacts at the dimer interface in a mechanism currently unique to Ste2.

There is currently only one high-resolution structure of a Class A GPCR dimer, the active state of the apelin receptor [52••]. This is different from dimers of Class C and Class D receptors as the interface is extremely small (140 Å^2^), comprising residues at the extracellular end of H3 (Figure 3a). Only one of the protomers is coupled to a G protein, and there are no contacts between the G protein and the adjacent protomer. Mutation of a key residue at the dimer interface (F101 A^3.24^) significantly reduced dimer formation and had a profound effect on the pharmacology of the apelin receptor, increasing basal activity and E_max_ significantly.

## Inactive GPCR structures by cryo-EM

The inactive state of GPCRs may only consist of 35–40 kDa of ordered protein, which is embedded in a detergent micelle typically ∼100 kDa in size and makes processing of cryo-EM images of these small membrane proteins highly challenging. To circumvent this problem, extra mass needs to be added to the receptor that can extend beyond the detergent micelle and facilitate particle alignment during image processing. An obvious solution is to repurpose successful strategies in engineering GPCRs for X-ray structure determination through either binding an antibody F_ab_ fragment [62], nanobody [63] or insert a small soluble protein such as BRIL in ICL3 [64].

**Figure 4:**
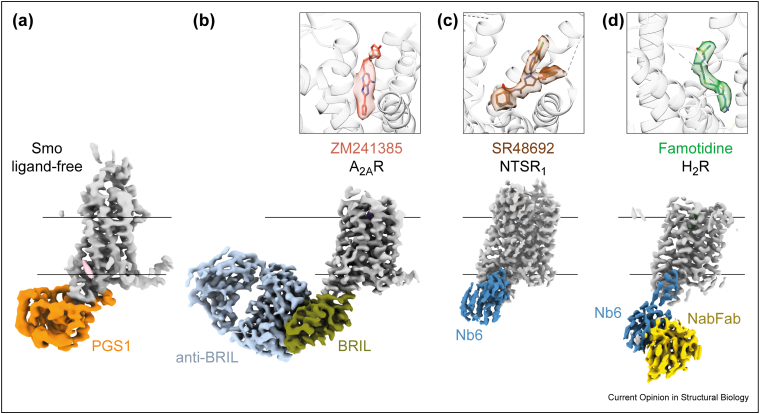
Examples of strategies to determine structures of GPCR inactive states. **(a)** Cryo-EM density of ligand-free Smoothened (EMDB-27062) [69]. **(b)** Cryo-EM density (EMDB-25648) of the adenosine A_2A_ receptor with a BRIL insertion in ICL3 and bound to an anti-BRIL Fab fragment [69]. **(c)** Cryo-EM density (EMDB-26589) of the neurotensin receptor NTSR1 engineered to contain the H5-ICL3-H6 region of MOR and bound to the anti-MOR nanobody Nb6 [67]. **(d)** Cryo-EM density (EMDB-26590) of the histamine H_2_ receptor engineered to contain the H5-ICL3-H6 region of MOR, bound to the anti-MOR nanobody Nb6 and the anti-nanobody F_ab_ (NabFab) [67]. Ligand density in the orthosteric binding pocket is shown above each receptor.

One recent approach was to graft a section of H5-ICL3-H6 from the mu opioid receptor (MOR) into a target GPCR and then bind nanobody Nb6 that specifically recognises this region [65,66]. This resulted in sub-3 Å resolution structures of the inactive states of NTS_1_R, H_2_R (Figure 4c,d) and somatostatin receptor 2 [67•]. Another approach was to insert BRIL in place of ICL3 in Frizzled5 and then use an anti-BRIL F_ab_/Nb complex to increase the mass further; the structure was determined by single-particle cryo-EM to 3.7 Å resolution, with the low resolution being explained by the flexibility of the GPCR-BRIL fusion points [68•]. This methodology was explored further [69•] to determine the structure of thermostabilised A_2A_R-BRIL bound to an anti-BRIL F_ab_ to 3.4 Å resolution (Figure 4b) and a Smoothened ICL3 chimera fused to *Pyrococcus* glycogen synthase (PGS) at 3.7 Å resolution (Figure 4a). A recent innovative strategy to create a three-point linkage between the heterodimer calcineurin and the β_2_AR facilitated the structure determination of the receptor either in the ligand-free state or bound to antagonist/agonist with overall resolutions between 3.5 and 3.9 Å [70•].

## Conclusions

The incredible advances in all the technology involved in single particle cryo-EM have made the structure determination of GPCR complexes in all conformational states considerably easier than using X-ray crystallography [71]. There are more advances in the cryo-EM pipeline and so the future holds rich promise for improving the throughput of GPCR structure determination, making it the premier tool for structure-based drug design and the determination of novel GPCR structures. A concerted effort over the coming years will undoubtedly determine structures of all human non-olfactory GPCRs.

## Declaration of competing interest

CGT is a shareholder and SAB member of Sosei Heptares. None of the other authors have any conflicts to declare.

## Data Availability

No data was used for the research described in the article.
